# ClPIF3-ClHY5 Module Regulates *ClPSY1* to Promote Watermelon Fruit Lycopene Accumulation Earlier under Supplementary Red Lighting

**DOI:** 10.3390/ijms23084145

**Published:** 2022-04-08

**Authors:** Tinghui Lv, Lili Zhao, Shuting Zhang, Jingyue Guan, Wei Liu, Hongyan Qi

**Affiliations:** 1 National & Local Joint Engineering Research Center of Northern Horticultural Facilities Design & Application Technology (Liaoning), Key Laboratory of Protected Horticulture of Education Ministry and Liaoning Province, College of Horticulture, Shenyang Agricultural University, Shenyang 110866, China; lvtinghui163@163.com (T.L.); vanbenben@163.com (S.Z.); gjy19990322@163.com (J.G.); 2 Institute of Vegetable Research, Liaoning Academy of Agricultural Sciences, Shenyang 110866, China; lili82sheng@sina.com (L.Z.); qinghuishuimu@126.com (W.L.)

**Keywords:** *Citrullus lanatus*, red light, lycopene, ClPIF3, ClHY5

## Abstract

Lycopene content is one of the important factors for determining watermelon fruit quality. In this study, a small-type watermelon was grown in a greenhouse with supplementary red lighting for 10 h per day. The results showed that the content of lycopene in the flesh was increased 6.3-fold after 25 days of supplementary red lighting. qRT-PCR analysis showed that *PHYTOENE SYNTHASE 1*(*ClPSY1*) is the major gene that responds to red light within the lycopene synthesis pathway. Moreover, we identified two key transcription factors that were involved in light signal transduction PHYTOCHROME INTERACTING FACTORS 3 (ClPIF3) and LONG HYPOCOTYL 5 (ClHY5) in watermelon flesh. The interaction experiments showed that ClHY5, a potent ClPIF3 antagonist, regulated *ClPSY1* expression by directly targeting a common promoter *cis*-element (G-box). Collectively, our findings identified that ClHY5 and ClPIF3 formed an activation-suppression transcriptional module that is responsive to red light and, through this model, regulated watermelon lycopene accumulation in greenhouse winter cultivation.

## 1. Introduction

Watermelon (*Citrullus lanatus* (Thunb.) Matsum. and Nakai) is one of the most economically important cucurbit crops worldwide. China is the largest watermelon producer, providing almost two-thirds of the world’s supply (http://www.fao.org/ (accessed on 9 March 2022)) [1]. In the cultivation of watermelon, the desired sunshine duration is more than 10 h per day, while the effective light time for winter cultivation in greenhouses is about 7 h per day, or even lower in northern China. Moreover, the light transmittance of the greenhouse in winter is about 50% due to the rain, snow, haze, or dust on the plastic film, thus leading to a decline in watermelon yield and fruit quality [2,3]. In recent years, with the advancements in light-emitting diode (LED) technology, the application of LED lighting in horticulture has increased dramatically [4,5]. Numerous studies have been conducted on the regulation of fruit quality, including anthocyanin, vitamin, secondary metabolites, and carotenoids, by LED lights [6,7,8,9,10]. Our previous study showed that supplementary red lighting improved melon quality [11].

Light is an important environmental factor for plant growth since it is not only an essential energy source for plants but also an important signal for plant growth and development [7,12]. PHYTOCHROME INTERACTING FACTORS (PIFs) are strongly regulated by light [13]. Red light induces photochrome (PHY) proteins to enter the nucleus in the form of active Pfr and promotes its interaction with PIF, resulting in PIF degradation through the 26S ubiquitin proteasome pathway [14,15]. However, PHYs exist in the cytoplasm as a non-bioactive Pr under dark conditions, which releases PIFs to interact with other genes to inhibit their transcription [16]. PIFs inhibit the transcription of target genes by binding G-box motifs in the promoter [17]. LONG HYPOCOTYL 5 (HY5) as a member of the basic leucine zipper (bZIP) transcription factor family and a central positive regulator in the light signaling pathway regulates the expression of thousands of genes [18]. HY5 directly regulates gene expression by interacting with ACE motifs, including Z-box (ATACGTGT), C-box (GTCANN), G-box, and mixed C/G (G) and C/A boxes [19,20]. PIFs and HY5 showed opposite accumulation patterns under light [19,21,22]. HY5 is also regulated by light, but in contrast to PIF, it accumulates under light [23,24,25]. For a broad range of responses, PIFs act antagonistically with HY5 in regulating gene expression [23,26]. Most of the studies on PIFs and HY5 were focused on seedlings and were shown to be less involved in the regulation of fruit color.

Flesh color is an important agronomical quality of watermelon. The carotenoid content determines the color of watermelon flesh [27]. As the main quality trait, the carotenoid biosynthetic and catabolic pathways were determined in watermelon. Phytoene synthase (PSY) is the first committed reaction of the carotenoid pathway. Phytoene desaturase (PDS) and ζ-carotene desaturase (ZDS) introduced four additional double bonds to producing phytofluene, ζ-carotene, and lycopene. During desaturation, ζ-Carotene isomerase (ZISO) and carotenoid isomerase (CRTISO) are required to catalyze the poly-*cis*-carotenoids to all-*trans*-carotenoids. Producing α-carotene by lycopene ε-cyclase (LCYE) and lycopene β-cyclase (LCYB) or β-carotene by lycopene β-cyclase (LCYB). 9-*cis*-epoxycarotenoid dioxygenases (NCEDs) are the precursors of abscisic acid (ABA) [28,29]. The red-fleshed watermelon mainly accumulates lycopene and a little β-carotene [27,30,31]. *ClPSY1* was reported to be responsible for the formation of lycopene content in watermelon fruit [27]. In plants carrying the *PSY1* knockout allele, lycopene content was reduced, showing fruit with yellow flesh and with no further change in color during ripening in tomatoes [32].

Lycopene is a precursor of the essential vitamin A and offers other health benefits due to its antioxidant properties [33]. In recent years, light regulation of lycopene synthesis was widely reported. However, the mechanism of light regulating watermelon fruit lycopene content has not been reported yet. In this study, we found that supplementary red lighting promoted lycopene accumulation earlier in watermelon flesh. Moreover, ClHY5 and ClPIF3 form a dynamic activation–suppression transcriptional module that regulates watermelon lycopene accumulation with supplementary red lighting under short and low light conditions. Low light reduces the fruit quality, while supplementary lighting improves fruit quality; this seems to be a common result but the mechanism behind this phenomenon has rarely been explored. In this research, we aimed to elucidate the molecular mechanisms regarding how red light regulates lycopene accumulation in watermelon flesh and hope to provide a theoretical basis for supplementary LED lighting in watermelon winter cultivation.

## 2. Results

### 2.1. Supplementary Red Lighting Promoted the Lycopene Accumulation Earlier in Watermelon Flesh

**Figure 1:**
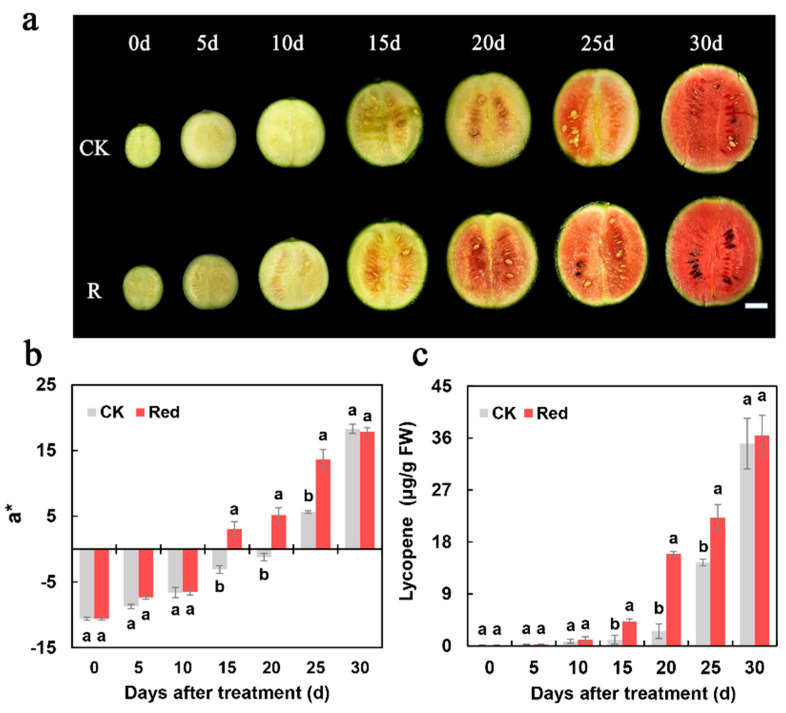
Flesh color and lycopene content in watermelon flesh after supplementary red lighting. (**a**) Flesh color of watermelon fruit during the fruit development stage (5 d, 10 d, 15 d, 20 d, 25 d, and 30 d after supplementary red lighting and natural light treatments). (**b**) Color difference analysis of flesh color using a chromatic aberration meter. (**c**) Lycopene content in watermelon fruit. Red: supplementary red lighting; CK: natural light (control). The data show mean values of three replicates (±SD) no less than three plants per replicate. The mean values of different letters were significantly different (*p* < 0.05). Scale bar: 3 cm.

The flesh color of cross-sections of watermelon fruit after the supplementary red lighting treatment at different development stages was observed. The results showed that the flesh color changed from green to red after 15 d of supplementary red lighting, and 5–10 d earlier than that under natural light (control) (Figure 1a). The change trends of the a* value (Figure 1b) and lycopene content (Figure 1c) of the flesh color were consistent with the flesh color appearance. After supplementary red lighting for 25 d, the content of the flesh lycopene was 15.91 μg/g FW, which was 6.3 times higher than the control. After supplementary red lighting for 15 and 25 d, the content of flesh lycopene was 4.24 and 1.54 times higher than the control. However, there was no significant difference with the control at 30 d. Additionally, we also measured the β-carotene content in fruit flesh and found that it increased significantly from 20 d to 25 d after supplementary red lighting relative to the control Figure S2).

### 2.2. Carotenoid Biosynthesis and Metabolism-Related Genes Expression after Supplementary Red Lighting

**Figure 2:**
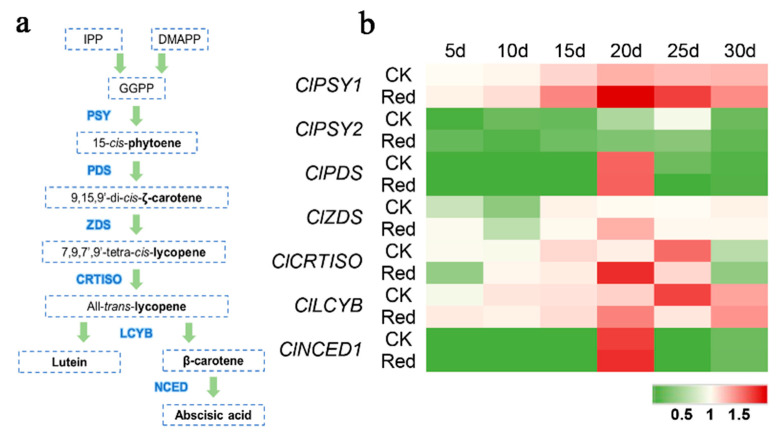
Relative expressions of carotenoid metabolism genes after supplementary red lighting. (**a**) Carotenoid metabolism pathway. (**b**) Changes in carotenoid metabolism genes expression level in watermelons after supplementary red lighting. Phytoene synthase 1 (*ClPSY1*); phytoene synthase 2 (*ClPSY2*); phytoene desaturase (*ClPDS*); zeta-carotene desaturase (*ClZDS*); carotenoid isomerase (*ClCRTISO*); lycopene β-cyclase (*ClLCYB*); 9-*cis*-epoxycarotenoid dioxygenase1 (*ClNCED1*). Red: supplementary red lighting; CK: natural light (control). The data show mean values of three replicates (±SD) with three plants per replicate. Green indicates a decrease and red an increase in gene expression (see the color set scale in the lower-right corner).

We used qRT-PCR to detect the expression level of genes related to carotenoid biosynthesis and metabolism (*ClPSY1*, *ClPSY2*, *ClPDS*, *ClZDS*, *ClCRTISO*, *ClLCYB*, and *ClNCED1*) (Figure 2a). During the synthesis of lycopene, we found that the expression level of *ClPSY1* was upregulated throughout the fruit development, and the expression level after the supplementary red lighting treatment was significantly higher than the control from 15 d to 25 d. However, there was no significant change in the expression level of *ClPSY2* during the fruit development and after the supplementary red lighting treatment. *ClPDS* was significantly upregulated at 20 d but there was no significant difference between the red lighting treatment and the control. *ClZDS* was significantly upregulated at 20 d relative to the control; however, its expression level was lower than *ClPSY1* (Figure 2b). During the synthesis of β-carotene, we found that *ClCRTISO* and *ClLCYB* were significantly upregulated at 20 d but significantly downregulated at 25 d after the supplementary red lighting treatment. *ClNCED1* had significantly upregulated expression at 20 d but there was no significant difference between the red lighting treatment and the control (Figure 2b). 

### 2.3. Phylogenetic and Expression Analysis of ClHY5

**Figure 3:**
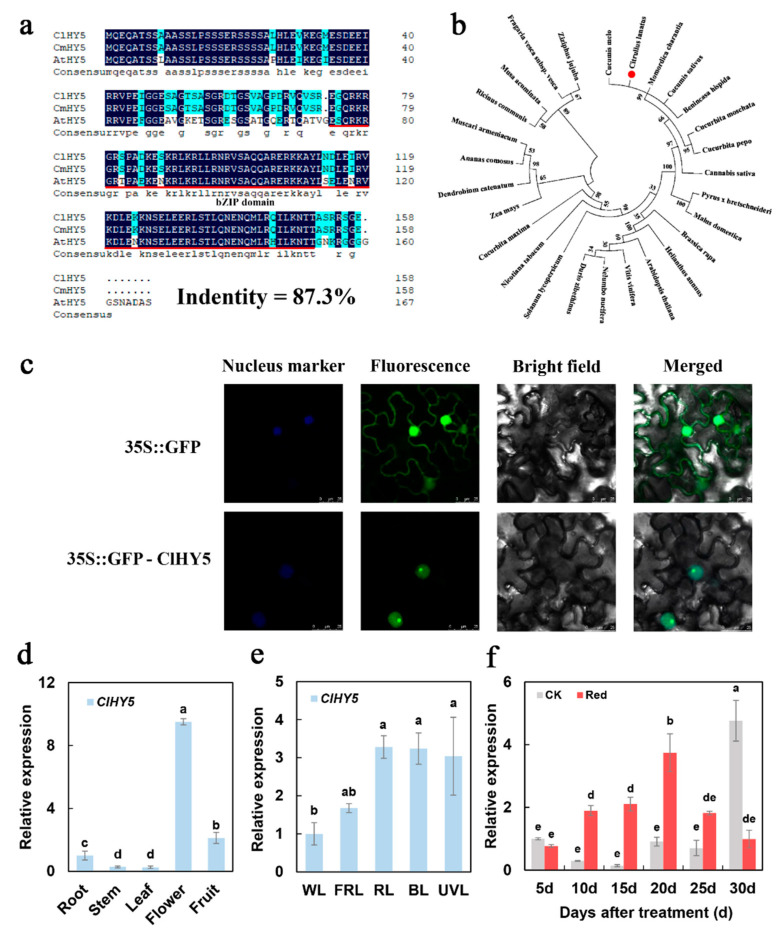
Sequence alignment, phylogenetic analysis, subcellular localization, and expression pattern of the *ClHY5* gene. (**a**) Amino acid sequence alignment of ClHY5, CmHY5, and AtHY5. The sequence in black shadow represents the same sequence and the red line is the bZIP domain. (**b**) Phylogenetic tree of ClHY5 with other HY5 proteins from different plant species. (**c**) Subcellular localization of ClHY5 in *N. benthamiana* leaf cells and the green dots are GFP fluorescent protein. Bars = 25 μm. (**d**) The expression levels of *ClHY5* in different tissues. (**e**) Responses of *ClHY5* in leaves to different types of light. WL, white light; FRL, far-red light; RL, red light; BL, blue light; UVL, UV light. (**f**) Response of *ClHY5* to supplementary red lighting treatment during fruit development. The data showed mean values of three replicates (±SD) with three plants per replicate. The mean values of different letters were significantly different (*p* < 0.05).

The *ClHY5* (Cla97C08G156620) sequence is from the Cucurbit Genomics Database (http://cucurbitgenomics.org/ (accessed on 11 March 2022)), which gives the full-length coding sequence (CDS) of *ClHY5*. Bioinformatics analysis found that ClHY5 contains 477 bp and encoded a 158-amino-acid protein (Figure S4). Protein functional domain analysis showed that the ClHY5 protein contained a typical leucine zipper structure (bZIP domain) at the C-terminal. There was a nuclear localization signal domain (NLS domain) at the N-terminal of the bZIP domain, which suggested that ClHY5 was a nuclear localization protein similar to AtHY5, a homolog of *Arabidopsis* thaliana. An alignment of the amino acid sequences of ClHY5, CmHY5, and AtHY5 revealed an 87.30% sequence identity (Figure 3a). Phylogenetic analysis showed that the ClHY5 and CmHY5, McHY5, BhHY5, and CsHY5 amino acid sequences were the most closely related, followed by other cucurbitaceae proteins, and then proteins from other species, consistent with their phylogenetic placement (Figure 3b). ClHY5 amino acid sequence analysis predicted that it localized to the nucleus (http://cello.life.nctu.edu.tw/ (accessed on 11 March 2022)) [34], and this hypothesis was tested via expressing the green fluorescent protein (GFP):HY5 fusion (ClHY5-GFP) driven by the constitutive CaMV 35S promoter in Nicotiana benthamiana leaves. The green fluorescent signal of ClHY5-GFP was only detected in the nucleus, while GFP alone was found throughout the entire cell (Figure 3c).

qRT-PCR analysis of *ClHY5* in different watermelon tissues revealed that it was universally expressed; the highest expression level was found in the flowers, and then in the fruit (Figure 3d). *ClHY5* responded to different light treatments in the leaves and had significantly upregulated expression after far-red light, red light, blue light, and UV-light treatments compared to the white light treatment (Figure 3e). During the fruit development stage, we found that the expression level of *ClHY5* was significantly increased after the supplementary red lighting treatment than the control from 10 d to 25 d (Figure 3f).

### 2.4. Phylogenetic and Expression Analysis of ClPIF3

**Figure 4:**
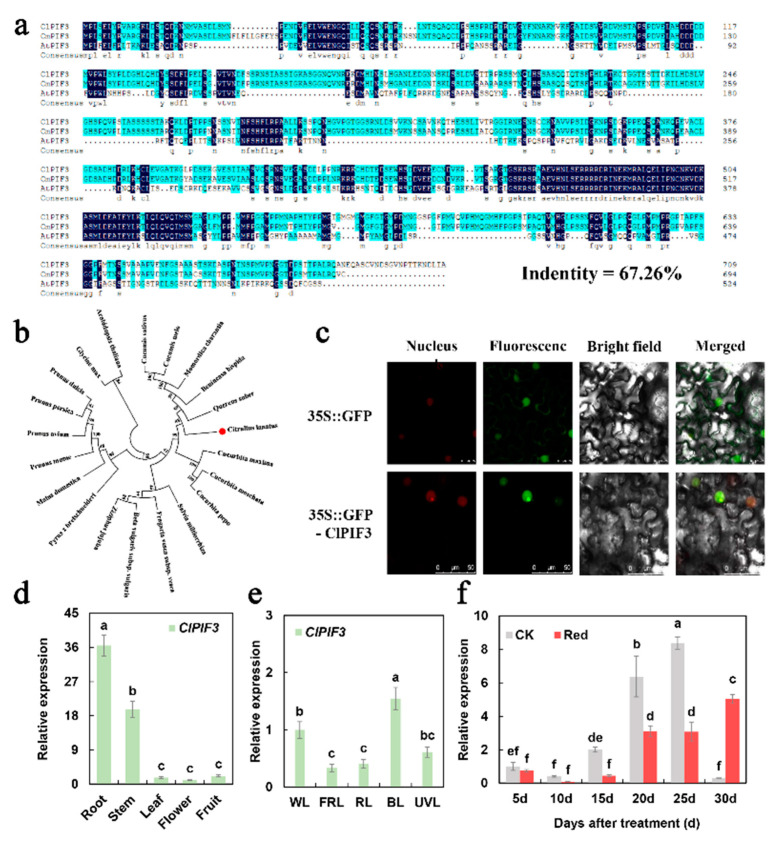
Sequence alignment, phylogenetic analysis, subcellular localization, and expression pattern of the *ClPIF3* gene. (**a**) Amino acid sequence alignment of ClPIF3, CmPIF3, and AtPIF3. The sequence in black shadow represents the same sequence (**b**) Phylogenetic tree of ClPIF3 with other PIF3 proteins from different plant species. (**c**) Subcellular localization of ClPIF3 in *N. benthamiana* leaf cells and the green dots are GFP fluorescent protein. Bars = 25 μm. (**d**) The expression levels of *ClPIF3* in different tissues. (**e**) Responses of *ClPIF3* in leaves to different types of light. WL, white light; FRL, far-red light; RL, red light; BL, blue light; UVL, UV light. (**f**) Response of *ClPIF3* to supplementary red lighting treatment during fruit development. The data showed mean values of three replicates (±SD) with three plants per replicate. The mean values of different letters were significantly different (*p* < 0.05).

The *ClPIF3* (Cla019998) sequence was from the Cucurbit Genomics Database (http://cucurbitgenomics.org/ (accessed on 11 March 2022)), which gives the full-length coding sequence (CDS) of *ClPIF3.* Bioinformatics analysis found that ClPIF3 contained 2133 bp and encoded a 711-amino-acid protein (Figure S4). Protein functional domain analysis showed that except for the loop region, ClPIF3 had conserved amino acid sites in other regions. An alignment of the amino acid sequences of ClPIF3, CmPIF3, and AtPIF3 revealed 67.26% sequence identity (Figure 4a). Phylogenetic analysis showed that the ClPIF3 and CsPIF3, CmPIF3, McPIF3, BhPIF3, and QsPIF3 amino acid sequences were the most closely related, followed by other cucurbitaceae proteins, and then proteins from other species, consistent with their phylogenetic placement (Figure 4b). ClPIF3 amino acid sequence analysis predicted that it localized to the nucleus (http://cello.life.nctu.edu.tw/ (accessed on 11 March 2022)) [34], and this hypothesis was tested via expressing the green fluorescent protein (GFP):PIF3 fusion (ClPIF3-GFP) driven by the constitutive CaMV 35S promoter in *Nicotiana benthamiana* leaves. The green fluorescent signal of ClPIF3-GFP was only detected in the nucleus, while GFP alone was found throughout the entire cell (Figure 4c).

qRT-PCR analysis of *ClPIF3* in different watermelon tissues revealed that it was universally expressed; the highest expression level was found in the roots and then in the stems and fruit (Figure 4d). *ClPIF3* responded to different light treatments in the leaves, and had significantly upregulated expression after blue light treatment and was significantly downregulated after far-red light and red light treatments compared with white light treatment (Figure 4e). We also found that the expression level of *ClPIF3* was significantly downregulated after the supplementary red lighting treatment relative to the natural light treatment from 15 d to 25 d (Figure 4f). 

### 2.5. ClHY5 Positively Regulated While ClPIF3 Negatively Regulated ClPSY1 Expression

**Figure 5:**
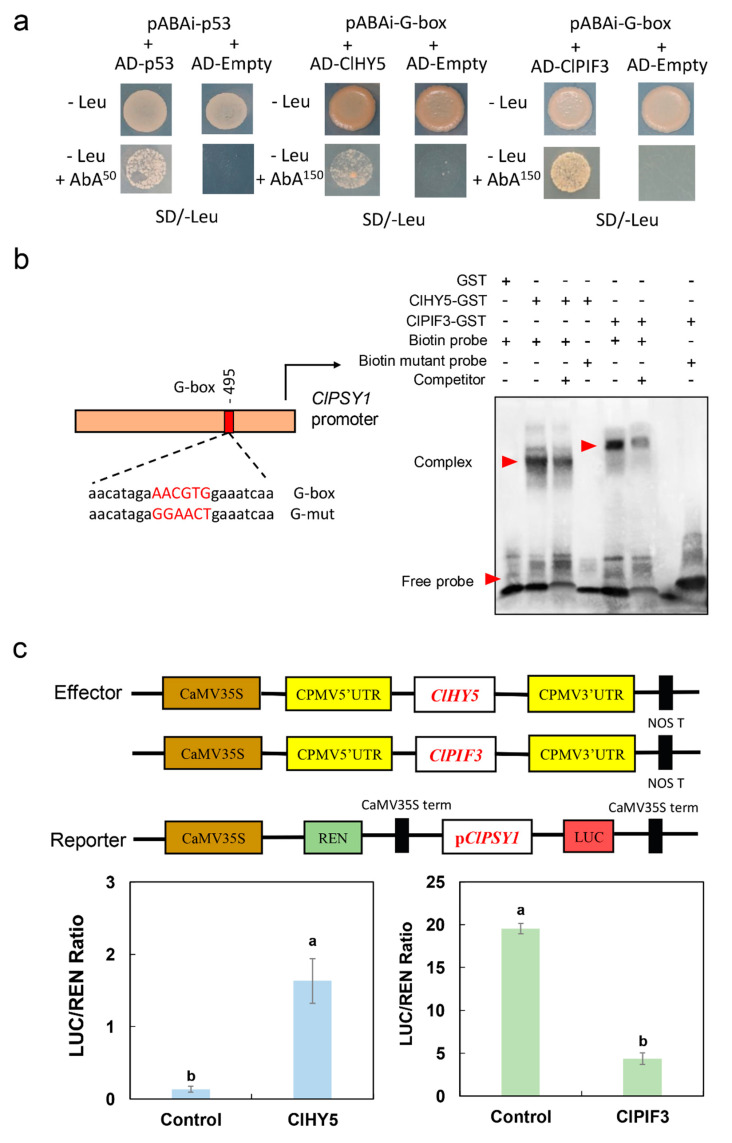
ClHY5 as a positive regulator and ClPIF3 as a negative regulator to regulate *ClPSY1* expression via binding promoter G-box *cis*-element. (**a**) Yeast one-hybrid (Y1H) assay showing that ClHY5 and ClPIF3 were bound to the G-box element of the *ClPSY1* promoter. The empty pGADT7-p53 and pAbAi-p53 vectors were used as the positive control, while the empty pGADT7 and pAbAi-G-box were used as the negative control. (**b**) Direct binding of ClHY5 and ClPIF3 to the G-box of the ClPIF3 promoter under in vitro conditions using an EMSA assay. ClHY5-GST: N-terminus of ClHY5 with GST-tag; ClPIF3-GST: N-terminus of ClPIF3 with GST-tag; Biotin probe: *ClPSY1* promoter with G-Box element biotin-labeled; Competitor: The probe which 100-fold higher concentration than biotin probe and with non biotin-labeled; Biotin mutant probe: *ClPSY1* promoter with mutant G-Box element biotin-labeled; The red arrows was the complex and free probe. (**c**) In vivo luciferase reporter assay showed that ClHY5 positively regulated and ClPIF3 natively regulated the *ClPSY1* expression. The ClHY5/ClPIF3 effector and the *ClPSY1* reporter were co-infiltrated into tobacco leaves, the luciferase signal was measured. Error bars for the dual-luciferase assays represent the SD of three independent experiments. The mean values with different letters were significantly different (*p* < 0.05).

We then verified whether ClHY5 and ClPIF3 directly regulated *ClPSY1* transcription in watermelon. First, the result of yeast one-hybrid (Y1H) showed that both ClHY5 and ClPIF3 could bind to the G-box element of the *ClPSY1* promoter in watermelon (Figure 5a). We further verified the binding activity with an electrophoretic mobility shift assay (EMSA). The results confirmed that ClHY5 and ClPIF3 could bind directly to the G-box of the *ClPSY1* promoter (Figure 5b). In addition, the ClHY5 and ClPIF3 that were individually driven by the 35S promoter were used as the effectors, and the *ClPSY1* promoter was then inserted into the pGreen-Ⅱ-0800-LUC vector (luciferase reporter vector) as a reporter in a transient tobacco leaf expression assay (Figure 5c). We found that ClHY5 positively regulated and ClPIF3 negatively regulated *ClPSY1* transcription in vivo (Figure 5c). In summary, ClHY5 and ClPIF3 could activate and inhibit *ClPSY1* transcription by directly binding to the G-box element on the promoter.

## 3. Discussion

Although previous research has advanced our understanding of light signal transduction, little is known about the molecular mechanism of red light in regulating watermelon flesh color. It is unknown whether using LED as an external light treatment affects plants through a photosynthesis pathway or light signal pathway; therefore, it is important to clarify this issue. In this study, we hoped to explore these mechanisms and provide some practical help for watermelon cultivation in winter

Flesh color is an important watermelon quality trait that is dependent on carotenoid synthesis during ripening [31]. Lycopene, which is a carotenoid, is found in the greatest amounts in red-fleshed watermelons [30]. Carotenoid content is affected by light in most higher plants. In fruit, red light increases the concentration of lycopene in tomato exocarp and the total carotenoid in citrus fruit. In leaves and stems of pea seedlings, carotene content was much higher in the red-light-treated group than the blue-light-treated group [7,35,36]. In our experiment, supplementary red lighting caused the watermelon flesh to change color earlier by promoting the lycopene accumulation under short and low light conditions (Figure 1). It is noteworthy that compared with the control, the lycopene content accumulated earlier under supplementary red lighting but there was no significant difference between the two treatments at harvest. Carotenoid biosynthesis is a large secondary metabolic pathway [37]. We speculate that lycopene biosynthesis in watermelon was not only induced by light but also regulated by many other factors. Though there was no significant difference in lycopene content in the watermelon flesh at the end of the treatment, the supplementary red lighting promoted lycopene accumulation earlier than the control, thus allowing the fruit to be harvested earlier; as such, it is a good choice for winter cultivation.

By analyzing the expression levels of the genes of carotenoid metabolism, *ClPSY1*, *ClCRTISO*, and *ClLCYB* were significantly differentially expressed between the two treatments (Figure 2). PSY is the first rate-limiting enzyme synthesized by lycopene, whose content determines the content of lycopene in the fruit [27]. CRTISO and LCYB are mainly responsible for the transformation and synthesis of β-carotene [27]. We speculate that the high level of *ClPSY1* expression promoted the downstream genes of the carotenoid metabolic pathway upregulation. It was reported that the high expression level of *ClLCYB* promotes lycopene catabolism [38]. This means that the high expression level of *ClLCYB* may result in low lycopene content. However, the relative expression of carotenoid metabolism genes showed that the expression level of *ClPSY1* was higher than that of *ClLCYB* (Figure 2b). The abundance of the synthetic gene was higher than the metabolic gene, which caused the lycopene content to increase. Therefore, we focused on the mechanism of *ClPSY1* expression regulated by red light. We cloned the *ClPSY1* promoter and found that there was a light-responsive G-box element at the location of 495 bp of the *ClPSY1* promoter (Figure S4).

To investigate the effect of light signals on lycopene accumulation in watermelon flesh, we identified two important transcription factors involved in the light signal transduction pathway. HY5 is a member of the bZIP transcription factor family that functions downstream of multiple families of photoreceptors. It promotes pigment accumulation in a light-dependent manner in *Arabidopsis* [39,40,41]. However, HY5 in watermelon has not been identified. In previous studies, ClHY5 and ClHYH (homologous gene) were identified in watermelon. qRT-PCR showed that *ClHY5* expression was significantly upregulated under supplementary red lighting (Figure S3). In this study, we found that the ClHY5 protein contained a typical bZIP domain and it had an obvious response to light. In the tissue-specific analysis, it was found that *ClHY5* in watermelon was highly expressed in the flowers (Figure 3). This was consistent with the expression pattern of *FaHY5* in strawberries [42]. The expression level of *ClHY5* was significantly higher than in the control at 10–25 days after red light treatment (Figure 3). This was consistent with the expression pattern of *ClPSY1* and suggested a tendency toward co-expression between them (Figure 2 and Figure 3). Therefore, we considered that the supplementation of red lighting can activate *ClHY5* expression and participate in carotenoid synthesis.

To explore the mechanism of lycopene accumulation under low light conditions, we identified that a basic helix–loop–helix transcription factor ClPIF3, which is a negative regulator of photomorphogenesis, played important roles in phytochrome signaling pathways [23]. As an inhibitor of light signal transduction, PIFs were found to be involved in plant stress resistance. Studies showed that PIF3 functions as a negative regulator of Arabidopsis freezing tolerance by directly binding to the promoters of C-REPEAT BINDINGFACTOR (CBF) genes to downregulate their expression [43]. However, there are few reports on ClPIF3 regulating watermelon fruit quality. In previous studies, ClPIFs (ClPIF1, ClPIF3, ClPIF4, ClPIF7) were identified in watermelon. qRT-PCR showed that *ClPIF3* expression was significantly downregulated under supplementary red lighting (Figure S3). In this study, *ClPIF3* expression was significantly downregulated under red and far-red light treatments compared with other treatments (Figure 4). 

It was shown that the protein stability of HY5 and PIFs were oppositely regulated by light [44]. Our results showed that the expression level of *ClPIF3* was significantly lower than that of the control during 10–20 days after red light treatment (Figure 4). This was in contrast with the expression pattern of ClHY5 (Figure 3). Red light had opposing effects on the *ClHY5* and *ClPIF3* levels (Figure 3 and Figure 4). Further, we established that ClHY5 promoted the expression of *ClPSY1* following exposure to red light, while ClPIF3 strongly suppressed its transcription in low light. Both PIFs and HY5 were reported to bind to G-box motifs to promote the expression of target genes at the transcriptional level [41,45]. In *Arabidopsis*, PIFs and HY5 regulate photosynthetic pigment accumulation using this model [40]. In this study, we found that both ClHY5 and ClPIF3 could bind to the G-box in the promoter of *ClPSY1* (Figure 5). Our data suggested an association showing that ClPIF3 and ClHY5 were able to bind to the *ClPSY1* promoter to antagonistically regulate *ClPSY1* expression to mediate lycopene accumulation in watermelon fruit.

**Figure 6:**
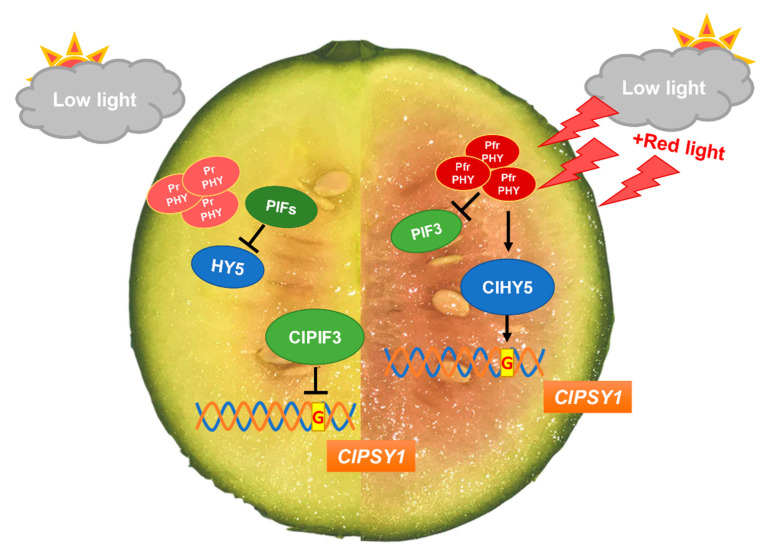
Model of ClPIF3 and ClHY5 cooperatively regulated the lycopene accumulated in watermelon flesh. Under low light, PHYs is present in the cytoplasm as inactive Pr form [47], PIFs protein abundance increased to promote the degradation of HY5 [48]. ClPIF3 inhibit *ClPSY1* transcription by directly binding to the G-box *cis*-element on the promoter to inhibited lycopene accumulation in watermelon flesh. Under supplementary red lighting, PHYs as active Pfr form, PHYs induces rapid in vivo phosphorylation of PIF3 preceding degradation [14]. Red light also induced PHYs to promotes accumulation of HY5 proteins [49]. ClHY5 activated *ClPSY1* transcription by directly binding to the G-box *cis*-element on the promoter to promoted lycopene accumulation in watermelon flesh.

PIF3 accumulates in the dark to promote dark development, and light induces rapid phytochrome-dependent phosphorylation, degradation, and deactivation [14,40,45]. HY5 protein accumulates in the light and is degraded in the dark [24,40,46]. Our results showed that the expression of ClPIF3 was significantly upregulated under low-light conditions, which inhibited the expression of *ClPSY1*. However, under supplementary red lighting, ClPIF3 was degraded and ClHY5 was accumulated to promote the expression of *ClPSY1* via binding promoter G-box *cis*-element. Taken together, we proposed a ClPIF3–ClHY5 module that mediated lycopene accumulation in watermelon flesh (Figure 6). These findings provide a new understanding that demonstrated that ClHY5 and ClPIF3 form an activation–suppression transcriptional module that responds to regulate *ClPSY1* transcriptional expression and indicates that supplementary LED lighting regulates lycopene accumulation through the light signal transduction pathway.

## 4. Materials and Methods

### 4.1. Plant Material, Growth Conditions, and Light Treatments

Cultivated small-type (<3 kg) watermelon (*Citrullus lanatus*) ‘Red xiaoyu’ (red-fleshed) was used and purchased from the Hunan Vegetable Research Institute (Changsha 410125, China). Watermelon seedlings were conventionally cultivated in the greenhouse until the second or third female flower was artificially pollinated, with one watermelon per plant kept.

The LED supplementary treatment started 10 days after pollination, with LED light irradiation occurring from 5:30 to 10:30 and from 14:30 to 19:30 for a total time of 10 h per day. The lamps were positioned (15 cm) directly above the watermelon fruit (Figure S1). Red LED lights with 14 W of power were used for supplementary light, where the wavelength was 660 nm. Supplementary red lighting (210 µmol·m^−2^·s^−1^) was applied under natural light conditions (R). Fruit grown under natural light alone was used as a control treatment (CK). The photosynthetic photon flux density (PPFD) during the treatment period was 400–500 µmol·m^−2^·s^−1^ on sunny days and 200–300 µmol·m^−2^·s^−1^ on cloudy days in the greenhouse. Samples were collected on days 0, 5, 10, 15, 20, 25, and 30 after supplementary red lighting, the peel and seeds were removed, and the flesh was frozen in liquid nitrogen and stored at −80 °C.

### 4.2. Flesh Color and Carotenoid Determination

The flesh color was measured using a Chroma Meter CR-400 (Konica Minolta, kobe, Japan) colorimeter at six points that were evenly selected around a lengthwise cut of the fruit, and the coordinates (a*) were recorded. 

Lycopene and β-carotene extraction were performed following a previous report with some modifications [50]. About 3 g of flesh was ground with liquid nitrogen and 20 mL absolute was added for the dehydration treatment, while 30 mL methyl alcohol was added in batches until the tissue turned white, at which point, the flesh residues were washed with 2% dichloromethane petroleum ether.

Lycopene and β-carotene measurements were performed following a previous report with some modifications [27]. The samples were dissolved in the mobile phase (2 methyl alcohol: 5 acetonitrile: 3 dichloromethane (*v*/*v*)) and filtered through a 0.22 µm organic nylon filter. High-performance liquid chromatography (HPLC) (Waters, e269, Milford, American) with a 4.6 × 250 mm 5-micron column (XDB-C18; Agilent, Palo Alto, California, American) was used for the analysis. The column temperature was 25 ℃ and the column flow rate was 1 mL/min^−1^. A wavelength of 502 nm was used for detection. The certified standard (Sigma, St. Louis, MO, USA) concentration was 0.2 mg/mL. Values were quantified using standard curves (y = 66.917x + 2.1408, R^2^ = 0.9992). All samples were analyzed in triplicate.

### 4.3. qRT-PCR Analysis

Total RNA extraction was achieved using an RNApure Plant Kit (CWBIO, Beijing, China). RNA samples (1 μg) were reverse transcribed into cDNAs using PrimeScriptTM RT Master Mix (Takara, China). SYBR Green PCR Master Mix was used for real-time quantitative PCR analysis (Takara, China). Primers were designed using Primer 5.0 software (Table S1). The relative expression was analyzed using the 2^−ΔΔCt^ method. All experiments were performed with three biological and technical replicates.

### 4.4. Phylogenetic Analysis

The ClHY5 (Cla97C08G156620) and ClPIF3 (Cla019998) amino acid sequences were obtained from the Cucurbit Genomics Database (http://cucurbitgenomics.org/ (accessed on 11 March 2022)) and the amino acid sequences of the other species protein were obtained from the NCBI nucleotide database (http://www.ncbi.nlm.nih.gov/nucleotide/ (accessed on 11 March 2022), Tables S3 and S4). MEGA 6.0 software was used to construct the phylogenetic tree using the neighbor-joining method, and the phylogenetic tree was constructed with 1000 bootstrap replicates to evaluate the reliability of the phylogenetic grouping. 

### 4.5. Subcellular Localization Analysis

The coding regions of *ClHY5* and *ClPIF3* were amplified using PrimeSTAR^®^ HS (Premix) (Takara, China) with appropriate primers (Table S1) and cloned into pCAMBIA1300-35S-GFP. We used pCAMBIA1300-35S-GFP containing *35S::GFP* (GFP) as a control. The two constructs and the P19 were transformed into the *Agrobacterium* GV3101 strain. Equal amounts of different combinations of Agrobacterium strains were mixed and injected into *N. benthamiana* leaves and GFP fluorescence was detected using a confocal fluorescence microscope after incubation at 24 °C for 72 h.

### 4.6. Yeast One-Hybrid Assays (Y1H)

The *ClHY5* and *ClPIF3* CDSs were amplified and inserted into the pGADT7 vector. The promoter sequences (3X-492 bp to −504 bp) of *ClPSY1* were amplified and inserted into the pAbAi vector. The primers are listed in Tables S1 and S2. The recombinant plasmid *ClPSY1*-pAbAi was transformed into Y1H to prepare the yeast strain. ClHY5-pGADT7 and ClPIF3-pGADT7 recombinant plasmids were respectively transformed into the prepared yeast strain to detect the interaction.

### 4.7. Electrophoretic Mobility Shift Assays (EMSA)

The ClHY5 and ClPIF3 CDS were cloned into the pGEX-6P-1 vector. Recombinant GST-ClHY5 and GST-ClPIF3 proteins were expressed in *Escherichia coli* strain BL21 and purified using a BeyoGold™ GST-tag Protein Purification Kit (Beyotime Biotechnology, Shanghai, China). The biotin-labeled probes from −487 bp to −509 bp of the *ClPSY1* promoter containing the ClHY5 and ClPIF3 binding site AACGTG and mutated probe GGAACT were commissioned by SBS Genetech (Beijing, China). The binding reaction was achieved using a Chemiluminescent EMSA Kit (Beyotime Biotechnology, catalog no. GS009).

### 4.8. Luciferase Reporter Assay (LUC)

The *ClPSY1* promoter was cloned into the pGreenII 0800-LUC vector. The coding regions of *ClHY5* and *ClPIF3* were cloned into pCAMBIA1300-35S-GFP. The recombinant plasmids were separately transformed into the *Agrobacterium* strain GV3101. An equal amount of different combinations of *Agrobacterium* strains were mixed and injected into *N. benthamiana* leaves. LUC and REN activities were detected using a Dual-Luciferase Reporter Assay system (Promega) after incubation at 24 °C for 72 h.

## 5. Conclusions

In this study, we found that supplementary red lighting promoted lycopene accumulation earlier in watermelon flesh than natural light (control). This made it clear that *ClPSY1* was the major gene that responded to red light in the lycopene synthesis pathway. By identifying ClPIF3 and ClHY5, we found that red light had opposing effects on these genes’ expression levels. In the end, we proposed a hypothesis stating that under a low light condition, ClPIF3 inhibited the expression of *ClPSY1* and prevented the accumulation of watermelon flesh color and supplementary red lighting decreased the expression of *ClPIF3* and increased the expression of *ClHY5* in watermelon fruits, thereby activating the transcription of *ClPSY1* and promoting the accumulation of lycopene.

Our results confirmed the effect of supplementary LED lighting in winter greenhouse cultivation, illustrated that providing supplementary red lighting regularly in the early stage of watermelon fruit enlargement could accelerate the color change through the ClHY5-ClPIF3 module, provided an effective mechanism to integrate light signals, and provided a theoretical basis for the early harvesting of watermelon under low light cultivation.

## Data Availability

Not applicable.

## Supplementary Materials

The following are available online at: https://www.mdpi.com/article/10.3390/ijms23084145/s1.
