# Supplementary material for: ClPIF3-ClHY5 Module Regulates ClPSY1 to Promote Watermelon Fruit Lycopene Accumulation Earlier under Supplementary Red Lighting
Source: Int J Mol Sci. 2022 Apr 8;23(8):4145. doi: 10.3390/ijms23084145 (PMC9024441; doi:10.3390/ijms23084145)
Supplement: Supplementary file 1 [file ijms-23-04145-s001.zip › Figure S4.pdf]

ClPSY1-promoter-CDS:
TTTCTTTACCAACTAAGTTAAAATTTGAGTGGAATATTGATGAATCTTTTGAAGAATTTT
AAGTATAATTTAGTTGATGAGACATATATACAAATTTTTTAGTTAAATAACTATATCCCCA
CCCATTTTATCTTTAAAAATGGTCCGAAATTTTTGGTTTTATTCATATGTTTGAAACTACA
TATTACTTAATTCATTGAAAAAAAAAAAACACATACTTAATTCAACAAATTAATTTTGATG
TTAAATATCGAATATTATTTAATTGAATAGTGTGCCATAATACTCAATTAATTAACATTGTT
AGTGTTCAAAAGAAAATTATGTACATAATACACAAACTTAAACTAACAATTTACTTGAA
ACTTAAATCCACTTGTTTAATATTCAAATAAATTAAATTTGAGCATTGTTCAAAAAATTA
AAAAAATGTAGGTTATTAGGTAAAAAGTTGAAGGTTCAAATTTACTTTGAGATTATTGT
GTCCAACATAGAAACGTGGAAATCAAAACTTTTTTTTAAGAAAAAAGAAACATAAAGT
TGAATGCCACATGTTTAGACCCATTTAGAAAACATTGAAATTCAAAACAATGGTGATCC
ACATTTGAAGTAATTGATTAAATTTATTCTTTTAAAGATTAACGGTGGAAGTTGATTTCT
ATATGGTTAATTGTTTGTTTGTTTTTTTTTCTTTTAAACTCAAACATTTCAATTGTGTGTA
TGCATGTCACATTTCACTACATCTTAAATAATGTTAAAATAAGTATAGAACTTATCTTATC
AACCTCGAGATTAGAGATTCAATTTTTCATGCTATTTATTATTAGAAAAAGAATCAGATT
AGTTGACCTATGATCAATGAATAACAGTTAATTACTTAACAATACTTCACCTTCCATGCA
TACCTTCTAATCACAAAGAAGAAAAAAACATTAAATTGCCAAAACAAAGGAGTTTGTT
AGTGATTGGGAGATTTGGGGTTCGAATCTTTCTAGTCTAAATATTAAAAGAAAGAAATA
TATATTAATTTTTTTAAAAGGACAAATATACAAAGTGACAAATTTGGACATTTAGGGAAA
ATTTCTTTTAACTCGAGATGGTATTTTTCTTAATTTATTTAGTGGATGAAAATTAAAAGA
GCTTAAATCCAAAAGCTATATTTAATTGGGCAAGAAACAATTCTGACACTCTTCTTTGT
CTCGGCTCCCATTCAAAAACAGAGCCGCAACTTGCATAAACTGGTTGTCGGGATCAAG
CAACCACACATTTTGCGGACCAAATTCTTCCAGCAACTACTAAGCTTCCATTAGAGCAA
CCTTCCACCACCAAACCCAAAAAGCGCACACCACCCTCAATTCAATTCCCCCACTAAA
AAGCACCCAATTCTATCAAACTTCAAACAGAGTCCCCCCNNNNNNNNNTGACCACAC
CCCCCCCCCCCCCCCCCCTTCCCTTGATCTCTCTGCTTCGTTGCTGTTCTTCAACAATCG
TAAGTGATTCTGTTCTTACATCATGTAATACCTTCCTGTTTTTGCTTTCCATTCTGGTATG
TTTCGTCAGATTCTGGGAGATTCGTTTCCTTTTTATCTTTAGTCAAAATTTCATGACGAA
AGAAACCGACCCTTTTCCACTTACCCCTCAAAAAGAGAAACAGTGAACTGAGCTTTCA
CATTTTCTGCCCTTTCTTGAGGATTATGATCATGTGGGTGTTTGGTATTTGTTGGTTATGC
TGTTTATTTTTATACCCACGCAATCGGGTTCTTATATTAATTTATTCTAGTCCATATCGGGC
GTATTTTTTTAAGTATATTTTTCGACGAATTACAGGCGCGATACAAAAGAGAAGCTAAG
TGGGAGTTTCCTCCGTGTCGAAGCATTGCTTAGAATTTCTTGGGCGGTTCAATTGGATT
TAAGTTGTTTTTTTCTTTTCTATTTCAATTTGCGACTGGGTTTCTCGATTCCTCTGCGATT
CGTTCACTAAAATTCTTCGAATCGATTGATA

ClHY5-CDS:

ATGCAGGAGCAAGCCACGAGTTCAGCCGCTGCTAGTTCTCTGCCTTCCAGCAGTGAAA
GATCCTCCAGCTCTGCTCTTCATCTCGAAGTTAAAGAAGGAATGGAGAGCGATGAGGA
GATCCGAAGAGTGCCGGAGATAGGCGGTGAATCGGCGGGAACATCCGCTTCCGGGAG
GGATACTGGTTCGGTTGCCGGTCCGGACCGGGTTCAAGTTTCTCGGGAGGGTCAAAGG
AAAAGAGGGAGAAGTCCGGCTGATAAAGAAAGCAAGAGACTGAAGAGATTGCTGAG
GAATAGAGTATCGGCACAGCAAGCGAGGGAGAGAAAAAAGGCATATTTGAATGACTTA
GAGATAAGGGTGAAGGATTTGGAGAAGAAGAACTCAGAACTTGAAGAAAGGCTTTCC
ACTTTACAAAATGAGAATCAGATGCTTAGACAAATTTTGAAGAACACAACGGCAAGTA
GGAGAAGTGGTGAGTGA

ClPIF3-CDS:

ATGCCTTTATCTGAGCTTTATCGTGTGGCCAGAGGGAAGCTTGATTCGACTCAAGACAA
GAACAACATGGTTGCTTCTGATTTATCTATGAATCCTGAGAATGACGTTTTCGAGCTGG
TGTGGGAGAATGGCCAAATTTTGCTGCAGGGCCAGTCCAATAGGACCAGGAAGTTAAA
CACTTCACAGGCTCAATGTTTACCATCTCATAGTCCCAGGGATAGAGATAGAGATGTAG
GATATTTCAACAATGCAAAGATGGTAAAGTTTGGGGCTATTGATTCTGTAGTAAGAGAT
GTTATGTCAACGGCCCCTTCGCCGGACGTCGAATTGGCTCATGATGATGATGATGATAT
GGTGCCTTGGCTAAGTTATCCTCTTGATGGACATCTGCAACACGATTATTCTTCTGATTT
CTTACCTGAATTATCTGGGGTCACTGTTAATGACTTTTCCTCACGCAATAGCATTGCATC
TTCTATAGGCAAAGCTAGTGGTGGTAATCAGGTAAACAGAGAGAAAGACATGCACCTA
AATTCTTTGCACGGTGCTAATCTAGAAGATGGCAATAATTCTAAGCTTTCTTCATTAGAT
GTTTCAACTACTAGACCTAGAAGTAGCATGAACCAATTACATTCATCAGCATCCCAGCA
AATCCAGACATCATTTCCACATCTCAGAACAAAATGCACTGGGGGCACTGAAAGTACT
ACAGACAAGATTCTCCATGATTCTCTTGTGGGCCACTCTCCTCAGGTTCCATCGATTGC
AAGCAGTTCTTCAAGCACAGCAAAGCAAAAGCTAGATCCAACTCCTCCAAGTAATTCA
TCTAATGTCATAAATTTCTCCCATTTTCTGCGACCTGCTGCTCTTCTCAAATCCAGTCCT
CAAAACCATGGTGTCCCTGGAACTGGAGGTTCGCGCAATTTGGACAGCGTGGTTAAGA
ACTGCTCGGCAGTTAATAAGCAAACCCATGAATCATCATTAATTGTAACTCGAGGTGGT
ATAAGAAATGAATCAAATTCATGTTGTAAAAATGCGGTAGTGCCTTCCATAGATGGTAA
AAATCCATCAGATGGCAAGCCTCCTGAGCAATCACAGGCTAATAAACAGCCTGAGGTT
GCTTGTTTAGGAGATTCAGCCGATCACGATGACCGCCTCAAACATTGTCTTGAAGTTGG
TGCGACAAAAGGATTGCCAGATAGTGAGAAGGGTGTTGAATCTATAATTGCCGCTTCC
GTTTGTTCACGGAATAGCGTGGAGGGAGCTTCTGATGATTTACCACCAAATAGGAAGA
GAAAATGTCATGACACTGAGGATTCTGAATGGCACAGTGATGATGTAGAAGAAGAATG
CAATGATGTGAAGAGAGTGACTTCTGCTCGAGGAACTGGTTCAAAAAGAAGTAGGGC
AGCAGAAGTGCATAATTTATCAGAAAGACGACGTAGAGATAGGATCAATGAGAAGATG
CGTGCACTGCAAGAGCTTATACCAAATTGTAATAAGGTCGACAAAGCTTCCATGCTTGA
TGAAGCCATTGAGTACTTGAAAACGCTTCAACTTCAGGTTCAGATTATGTCAATGGGA
GCTGGTTTGTTCATGCCTCCTGTGATGTTCCCCGGAGGAATGCCACCCATGAATGCACC
CCACATTTACCCACCCATGGGTATAGGAATGGGAATGGGAATGGGATTTGGGATAGGTA
TGCCAGACATGAATGGTGGTTCTCCTGGTTTTCCCATGGTTCAAGTGCCACACATGCAA
GGAATGCATTTCCCCGGCCCATCTATACCAGCTCAAACTGTAATGCATGGATTGCCAAG

TTCTAACTTTCAGGTTCTTGGACTTCCTGGTCAAGGACTTCCCATGCCCATGCCACGTG
GACCGATTGCCCCATTTTCTGGAGGGCCTTTTATGACTAATTCCAGTGTGGCAGCAGCT
CCTGTGGAGAATTTTGGTTCAGCTGCAGCCTCTACCTCGAAAGATGCATCTCCAAACAT
TAATTCACCCATGGTGCCAAATGGTGGCACCGACCCTTCAATTACTCCAGCTCTTAGAC
AGGCTAATGAACAAGCTTCCTGTGTTAATGACAGCGGTGTGAATCCCACCACCAAAAA
CGACCTTATAGCTAACTAA
