# Supplementary figures and images for: ClPIF3-ClHY5 Module Regulates ClPSY1 to Promote Watermelon Fruit Lycopene Accumulation Earlier under Supplementary Red Lighting
Source: Int J Mol Sci. 2022 Apr 8;23(8):4145. doi: 10.3390/ijms23084145 (PMC9024441; doi:10.3390/ijms23084145)

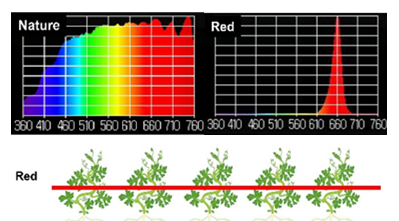

Supplement: Supplementary file 1 [file ijms-23-04145-s001.zip › Figure S1.tif]

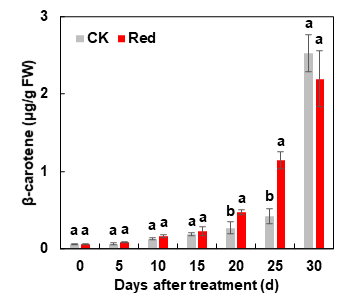

Supplement: Supplementary file 1 [file ijms-23-04145-s001.zip › Figure S2.tif]

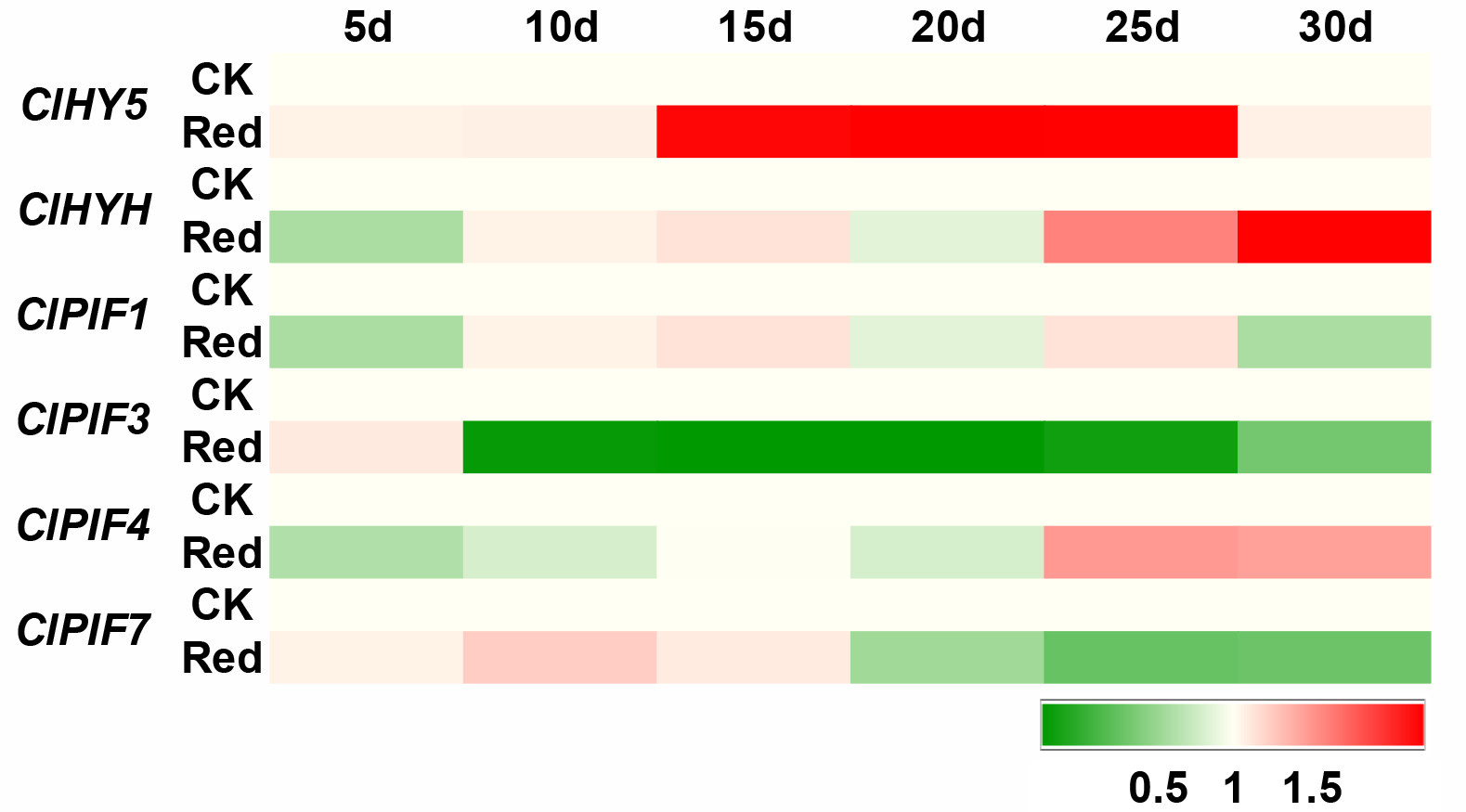

Supplement: Supplementary file 1 [file ijms-23-04145-s001.zip › Figure S3.png]
